# Characterization of Potential Risk Pathways for African Swine Fever Introduction Into Brazil

**DOI:** 10.1155/tbed/4187659

**Published:** 2026-05-21

**Authors:** Isadora Martins Pinto Coelho, Érica Lorenza Martins Araújo, Breno Oliveira Lima Ramos, Ailton Junior Antunes da Costa, Lorena Diniz Macedo Silva Maia, Ana Luisa Martins Brum, João Paulo Amaral Haddad, Rafael Romero Nicolino

**Affiliations:** ^1^ Veterinary School, Preventive Veterinary Medicine Department, Universidade Federal de Minas Gerais, Belo Horizonte, Brazil, ufmg.br

**Keywords:** African swine fever, immigrant workers, migration flows, seizure of animal products, surveillance, swine industry

## Abstract

African swine fever (ASF) poses a serious threat to global swine production, and its reintroduction into Brazil, the world’s third‐largest pork exporter, would have severe socioeconomic consequences. This study characterized the potential risk pathways for ASF introduction in Brazil by analyzing three key aspects: seizure of informally imported animal‐origin products (AOP) at airports, entry of individuals from ASF‐affected countries into Brazil, and the profile of immigrant workers. A total of 4301.38 kg of AOP from ASF‐affected countries, mainly China, Italy, Nigeria, and Germany, were seized at Brazilian airports in 2023. The most common nationalities involved were Chinese and Brazilian. Patterns of traveler entry from ASF‐affected countries revealed relationships among border entry points, visa types, and continent of origin. Seasonal patterns of international passenger arrivals were significantly correlated with the average seasonal index (ASI) of ASF outbreaks in Europe (Spearman *r* = 0.69, *p* < 0.05) and Asia (*r* = 0.62, *p* < 0.05), with both increasing during periods of intensified human mobility, such as holiday seasons, suggesting that human movement may represent one of several factors contributing to this seasonal alignment. Furthermore, the occupational and spatial profiles of immigrant workers from these countries showed that many were employed in roles involving direct contact with livestock and lived in states with high pig densities. These findings provide crucial evidence for implementing risk‐based surveillance. Additionally, the results underscore the importance of targeted educational measures for immigrant workers involved in livestock activities, aiming to mitigate the risk of ASF introduction and spread in Brazil.

## 1. Introduction

Globalization has intensified the movement of people and goods across countries, resulting in growing concerns about sanitary risks associated with the importation of pathogenic agents [1]. With respect to animal health, there is particular concern about the introduction of diseases with a major impact on swine production, such as African swine fever (ASF), classical swine fever, and foot‐and‐mouth disease (FMD) [2, 3], mainly through animal‐origin products (AOP) and fomites [4, 5]. In this context, the introduction of ASF into new areas through AOP has been widely reported in the literature [6–13]. Due to increasing concerns about the global introduction and spread of ASF, efforts have intensified, particularly in the surveillance of swine products and by‐products at borders [14, 15].

ASF is a highly contagious viral disease affecting both domestic and wild pigs [16]. Since its global re‐emergence and spread across Europe, Asia, and, more recently, the Americas, the disease has raised growing concerns regarding the potential introduction of the virus into ASF‐free regions [17]. The etiological agent, ASF virus (ASFV), is a large double‐stranded DNA virus belonging to the family Asfarviridae [18]. One characteristic that makes ASFV particularly difficult to control is its high environmental stability [19]. ASFV can persist for extended periods in contaminated materials, including meat and processed pork products, as well as fomites such as clothing, equipment, and vehicles [3]. In addition, the virus can remain viable for months in refrigerated or frozen pork products, which increases the risk of long‐distance dissemination through the movement of contaminated AOP and materials [20].

These biological characteristics are directly related to the mechanisms through which the virus may spread across regions and countries [4, 5, 21]. In the context of transboundary animal diseases, a pathway refers to the sequence of processes or mechanisms through which a pathogen may move from an infected source area to a susceptible host population [22]. For ASF, these pathways often involve the movement of infected animals, contaminated AOP, fomites, or infected materials transported by people, cargo, or equipment [4, 5]. Understanding and characterizing such pathways are essential for designing effective risk‐based surveillance and prevention strategies [22].

Within this global context, Brazil has also faced challenges related to ASF. The first outbreak in the country occurred in 1978, after an airport employee fed his pigs with food waste from international flights [8, 23, 24]. Brazil has been considered ASF‐free since 1984 and currently holds a prominent position in the global swine industry, ranking as the fourth‐largest producer and third‐largest exporter of pork worldwide [25]. Consequently, the reintroduction of the disease poses a serious socioeconomic threat. Given Brazil’s sanitary status and its economic relevance in the global pork market, assessing the potential risk of ASF introduction into the country has become a priority for animal health authorities.

In response to the global spread of ASF in recent years, several studies have evaluated the risk of virus introduction into ASF‐free regions [26–28]. In 2022, the Food and Agriculture Organization of the United Nations (FAO) conducted a qualitative risk assessment to identify the highest‐risk pathways for ASFV entry from the Dominican Republic and Haiti into countries and territories in the Americas that remain unaffected [28]. This study will build on FAO’s risk assessment to better characterize potential pathways for ASF introduction into Brazil. Within this framework, the introduction of ASF via the formal importation of AOP was considered to be negligible to low risk, with low uncertainty. In contrast, the informal importation of AOP is described as one of the highest‐risk pathways for ASF introduction into Brazil, with the risk classified as low‐to‐moderate and high uncertainty. Such movements can occur through multiple routes, including postal shipments, items carried in passenger luggage, and border crossings (e.g., on foot or by vehicle) [28]. However, due to the informal nature of this movement, data on AOP transit are scarce. Nevertheless, seizure records of such products may help to construct a profile of the main stakeholders involved in these practices, enabling a better understanding of volumes and frequencies [28].

Building on this perspective, behavioral factors have become particularly relevant. Individuals visiting their country of origin have stronger incentives compared to tourists to return with food from those locations, including AOP [28]. Given this scenario, understanding the profile of immigrants residing in Brazil, particularly those originating from ASF‐affected regions, becomes relevant, as such data may indicate potential demands for AOP from these areas [26]. Another point of concern, according to FAO’s analysis, is the introduction of the virus into Brazil through fomites, classified as the highest‐risk pathway among those described, with a moderate risk and high uncertainty. Fomites include a wide variety of materials, equipment, and clothing, and the virus can survive for weeks on these objects, particularly if individuals have recently visited pig farms [28]. This risk highlights the importance of understanding the dynamics of human movement across borders, including places of origin, border entry, route of entry (air, land, or sea), seasonality, and the type of visa granted. Moreover, the risk of fomite‐mediated introduction highlights the importance of profiling immigrants residing in the country.

Although previous international risk assessments, such as the FAO analysis [28], have identified the main potential pathways for ASF introduction into countries in the Americas, these assessments were conducted at a broad regional scale and do not provide a detailed empirical characterization of how these pathways are structured in the Brazilian context. In particular, limited information is available regarding how patterns of informal AOP movement, international passenger flows, and the occupational profiles of immigrants from ASF‐affected countries may interact to influence the risk of virus introduction.

Therefore, the objective of this study is to characterize key elements associated with potential pathways for ASF introduction into Brazil by analyzing three complementary dimensions: (i) seizures of informally imported AOP at Brazilian borders, (ii) patterns of human mobility entering the country, including border entry profiles and flight seasonality, and (iii) the occupational and spatial profile of immigrant workers from ASF‐affected countries. By integrating these elements, this study aims to identify stakeholders, temporal patterns, and contextual factors associated with potential ASF introduction pathways, providing evidence to support risk‐based surveillance and prevention strategies in Brazil.

## 2. Material and Methods

**Figure 1:**
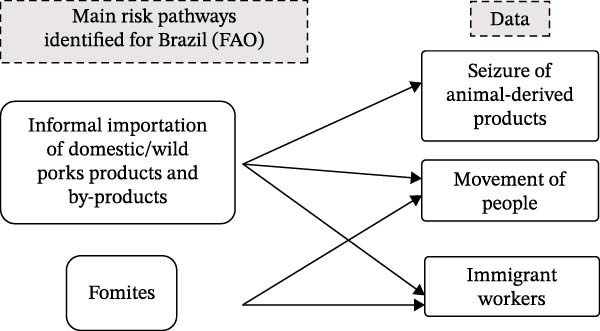
Data used according to the risk analysis conducted by FAO.

This study included three analytical components to characterize the risk of ASF introduction into Brazil (Figure 1).

All analyses were restricted to countries that reported ASF outbreaks between 2018 and 2022 according to FAO reports (Table S1).

### 2.1. Seizure of Informally Imported AOP

For the profile of informal AOP seizures at Brazilian borders, data from the Ministry of Agriculture and Livestock (Ministério da Agricultura e Pecuária—MAPA) were used, available nationwide for 2023 and covering traveler baggage at border entry points (road and air transport). The seized products were presented in quantity (kilograms) and relative frequency, segmented by product type, month, state, surveillance unit of seizure, as well as the traveler’s nationality and the country of origin of the seized product. The timeframes considered for each dataset were determined by the availability of complete and consolidated data from the respective institutions.

### 2.2. Movement of People—Profile of Border Entry

For the analysis of the profile of border entry into Brazil, data from the International Traffic System of the Ministry of Justice and Public Security (Sistema de Tráfego Internacional—STI), available and complete for the year 2022, were used. The variables analyzed were visa type, border of entry, traveler nationality, municipality and state of migration, and continent of origin. Two different variables were available in the dataset to describe the travelers’ visa information. The first, “Visa Type,” includes broad categories (Brazilian, tourist, transit, resident, temporary, border, unspecified, and nonnationals). In addition, a second variable provided more detailed classifications (e.g., tourism, air crew, maritime crew, business visit, maritime crew audit, and consulting), which allowed a more refined characterization of travel purposes in the analyses.

The results were presented in terms of frequency, highlighting the most common border entry points, as well as the visa types associated with them. Multiple correspondence analysis (MCA) was performed using the variables continent, border entry, state, and visa type to explore the relationship among categorical variables [29, 30].

### 2.3. Movement of People—Flight Flows

For the analysis of flight flows related to border entry, data from the National Civil Aviation Agency (ANAC) covering the period 2000–2022 were used, restricted to flights with Brazil as the destination country. The time‐series analysis considered data up to 2019 to minimize the influence of COVID‐19 in international travel patterns. Moving averages (order = 12 months) were calculated, followed by the average seasonal index (ASI), as described by Coelho et al. [31]. The Spearman correlation test (Spearman, 1904) was then used to assess the association between the ASI of ASF outbreaks in Europe and Asia and the ASI of international passenger arrivals from these continents into Brazil. For this analysis, ASF outbreaks reported in domestic pigs and wild boar were considered jointly to represent the overall seasonal pattern of ASF occurrence. Comparative plots were produced to visually explore the alignment between the seasonal patterns of ASF outbreaks and international passenger flows.

### 2.4. Immigrant Workers

To illustrate the profile of immigrants with formal employment ties originating from ASF‐affected countries, data on the admission of immigrant workers into the Brazilian formal labor market, as reported on the Labor Immigration Portal, were analyzed for the period 2021–2023. The variables used were nationality of origin, state and municipality of destination, and job category. For the animal health risk profile, subclasses of economic activity, defined by the National Classification of Economic Activities (CNAE/IBGE), were selected that could potentially allow contact between workers and livestock species, totaling 55 categories (Table S2). Occupations not included were grouped into the category other. For presentation and discussion purposes, categories were reclassified into the following: possibility of direct contact with animals, trade of AOP and by‐products, transport and cargo, slaughterhouses, and others.

Descriptive and analytical statistics were performed in R software (Version 4.3.0) [32]. The packages used include: “arrow” [33], “dplyr” [34], “ggplot2” [35], “ggpubr” [35], “lmtest” [36], “forecast” [37], and “trend” [38]. For line charts in the time‐series analysis of flights, the autoplot function was applied. Seasonality analyses and the construction of general seasonality and subseries plots were carried out using the functions ggseasonplot, ggsubseriesplot, and boxplot.

Two thematic maps were created using QGIS 3.32 [39] to illustrate the spatial distribution of immigrant admissions from ASF‐affected countries into the Brazilian formal labor market between 2021 and 2023. The first map displays the categories of sanitary relevance for animal health according to the state of destination and also includes the swine population of each state to provide context on the potential exposure risk. The second map illustrates categories involving workers with direct contact with animals based on the most frequent destination municipality.

## 3. Results

### 3.1. Overview

#### 3.1.1. Seizure of Informally Imported AOP

In 2023, a total of 32,490.62 kg of informally imported AOP were seized at Brazilian borders, of which 57.43% corresponded to “Meat and sausages” (18,658.58 kg). In total, 5989 seizures were recorded, with 65.67% at airport entries (3933) and 34.33% on land borders (2056). The month with the highest number of seizures was March, accounting for 11.54% (691) (Table S2). The frequency of seizures by country of origin of the products is presented in Table S3.

Given the main objective of this study, which is the risk associated with the introduction of ASF, the subsequent results describe only seizures reported in countries that reported ASF outbreaks between 2018 and 2022, as reported by the FAO.

In 2023, 4301.38 kg of AOP originating from ASF‐affected countries were seized, representing a total of 1046 seizure events. Of this amount, most came from China (37.69%, *n* = 395), Italy (24.43%, *n* = 256), Nigeria (17.18%, *n* = 180), Germany (4.39%, *n* = 46), South Korea (2.86%, *n* = 30), and Haiti (2.77%, *n* = 29). The quantity of AOP seized by the country of origin is shown in Table 1.

**Table 1:** Number of AOP seizures in 2023 from ASF‐affected countries in recent years, according to the month of seizure.

Month	Seizures	Percentage (%)
January	43	4.11
February	78	7.46
March	88	8.41
April	121	11.57
May	117	11.19
June	120	11.47
July	92	8.79
August	128	12.28
September	84	8.03
October	112	10.71
November	5	0.48
December	58	5.54
Total	1.046	100

Of these products, 27.81% (1196.40 kg) belonged to the category “Meat and sausages.” Among the total seizures of “Meat and sausages,” 40.55% (485.11 kg) originated from China, 35.17% (420.79 kg) from Italy, and 5.32% (63.62 kg) from Germany (Table 2).

**Table 2:** Amount of meat and sausages seized (kg) in 2023 according to the ASF‐affected country of origin.

Country of origin	Quantity (kg)	Percentage (%)
South Africa	10.9	0.91
Germany	63.62	5.32
Belgium	14.26	1.19
Belarus	1.05	0.09
Bulgaria	3.14	0.26
Chad	0.1	0.01
Camboja	1.33	0.11
China	485.11	40.55
South Korea	25.12	2.09
Croatia	3.95	0.33
Slovakia	0.48	0.04
Philippines	14.43	1.20
Greece	0.3	0.02
Haiti	10.8	0.89
Hungary	4.6	0.38
India	9.8	0.82
Indonesia	6.74	0.56
Italy	420.79	35.17
Malaysia	1.42	0.12
Myanmar	1.23	0.10
Nigeria	33.42	2.79
Poland	40.57	3.39
Dominican Republic	5.14	0.43
Russia	14.4	1.20
Serbia	2.4	0.20
Thailand	9.07	0.76
Ukraine	2.75	0.23
Vietnam	19.49	1.63
Total	1196.40	100

Regarding the nationality of travelers involved in the seizures, 39.90% (477.38 kg) of the seized meat and sausages were carried by Chinese, 22.25% (266.04 kg) by Brazilians, and 18.56% (222.06 kg) by Italians (Table 3). Some profiles are worth highlighting. For instance, in the case of meat and sausages originating from Haiti and the Dominican Republic, 100% of those were carried by Haitians and the Dominican Republicans. For China, the profile was similar, as almost all AOP seized indeed belonged to Chinese. However, in the case of seizures originating from Italy and Germany, approximately half of the AOP were seized from Brazilians.

**Table 3:** Amount of meat and sausages seized (kg) in 2023 according to the nationality of the traveler (originating from ASF‐affected countries).

Nationality	Quantity (kg)	Percentage (%)
China	477.38	39.90
Brazil	266.04	22.24
Italy	222.06	18.56
Poland	29.75	2.49
Nigeria	28.66	2.40
South Korea	25.12	2.10
Germany	24.91	2.08
Vietnam	19.36	1.62
Philippines	14.43	1.21
Russia	14.4	1.20
Haiti	10.8	0.90
Belgium	7.95	0.66
Thailand	7.88	0.66
Indonesia	6.74	0.56
South Africa	5.8	0.48
Slovakia	0.37	0.03
Australia	0.3	0.03
Bangladesh	5.3	0.44
Dominican Republic	5.14	0.43
India	4.5	0.38
Romania	3.48	0.29
Bulgaria	3.14	0.26
Ukraine	2.75	0.23
Bolivia	2.03	0.17
Malaysia	1.42	0.12
Cambodia	1.33	0.11
Myanmar	1.23	0.10
Belarus	1.05	0.09
Switzerland	0.92	0.08
Argentina	0.76	0.06
Portugal	0.76	0.06
United States	0.68	0.06
Total	1196.40	100

Regarding the surveillance units, Guarulhos International Airport (São Paulo [SP]/VIGI‐GRU) accounted for the highest number of seizures, representing 62.51% of the total (613.32 kg), followed by Tom Jobim International Airport (Rio de Janeiro [RJ]/VIGI‐GIG) with 31.85% (312.54 kg). The quantity and percentage of products seized by the surveillance unit can be found in Table S4.

#### 3.1.2. Profile of People Crossing Brazilian Borders

In 2022, a total of 9.3 million people entered Brazil through border checkpoints. The continent of origin for most of these individuals was South America, accounting for 79.53% (7,396,671), followed by Europe 10.79% (1,003,675), North America 4.93% (459,303), Asia 3.26% (303,282), Central America and the Caribbean 0.7% (64,824), Africa 0.68% (63,552), and Oceania 0.18% (17,076). Regarding the analysis filtered by ASF‐affected countries, the highest frequency of entries originated from Europe (56.71%, 319,350), Asia (37.97%, 213,843), Africa (3.01%, 16,970), and Central America and the Caribbean (2.3%, 12,979) (Table S5).

It is essential to note that the distribution by border entry differed between the overall and ASF‐specific analyses. Considering only European countries with ASF outbreaks, a higher proportion of air entries was observed (83.71%, 267,319) compared to the general proportion (64.27%). Conversely, for Asian countries, 59.48% (127,196) of travelers from ASF‐affected countries entered Brazil by sea, contrasting with the overall maritime proportion of 27.34% (153,947) (Table S5). These countries also showed a higher proportion of river entries (8.39%, 17,934) compared to the general rate (4%, 22,530) (Table S5).

The vast majority of European travelers entered Brazil by air, with notable percentages from Germany (88.70%, 104,214), Belgium (89.79%, 15,859), Belarus (86.98%, 1022), and Italy (93.08%, 101,327) (Table S6). However, some countries displayed a different profile, such as Ukraine, with 32.36% (5960) entering by air and 53.35% (9826) by sea (Table S6).

For Asia, although the general proportion was higher for sea entries, it is noteworthy that Thailand, Malaysia, Nepal, Mongolia, Bhutan, and Cambodia showed predominantly air migration profiles to Brazil, corresponding to 78.17% (2585), 78.20% (2267), 96.73% (1125), 90.32% (140), 100% (53), and 90% (27) of each country’s total, respectively (Table S6). Regarding Central American countries, both Haiti and the Dominican Republic displayed predominantly air entry profiles, 96.52% (6675) and 89.00% (5396), respectively. The Dominican Republic also showed a noticeable proportion of land travelers (565) (Table S6).

The main nationalities crossing Brazilian borders in 2022 from ASF‐affected countries were Germany, 20.86% (117,485), the Philippines, 19.56% (110,132), Italy, 19.33% (108,858), India, 9.21% (51,886), and China, 5.8% (32,644).

Table 4 presents the number of people entering Brazil by visa type. Although the “Tourist” visa was one of the most relevant categories in both analyses, it is important to note that while the “Transit” visa accounted for 7.66% in the general analysis, this classification reached 42.15% in the ASF‐specific analysis.

**Table 4:** Classifications of visa type for the entry of people into Brazil (general analysis and ASF‐affected countries analysis).

Visa type	Total	Percentage (%)	Countries with ASF outbreaks 2018–2022	Percentage (ASF) (%)
Brazilian	5,190,484	55.76	31,979	5.68
Tourist	2,684,364	28.84	191,495	34
Transit	712,635	7.66	237,392	42.15
Resident	425,884	4.58	54,000	9.59
Temporary	294,088	3.16	48,275	8.57
Border	943	0.01	11	0
Unspecified	31	0	3	0
Nonnationals	16	0	4	0

The descriptive analysis of visa types by nationality is presented in Table S7. This table shows that European countries have a higher percentage of tourist classifications, whereas Asian countries have a relevant proportion of transit classifications. African countries and Haiti present a considerable percentage of temporary and resident visas. In the case of the Dominican Republic, the largest proportion corresponds to the tourist visa.

Based on the more detailed visa classification variable, which breaks down travelers’ visa information into specific categories, the most frequent categories were tourism (63.10%, 2,684,364), air crew (7.78%, 330,936), resident (6.93%, 294,830), and business visit (5.37%, 228,309). When filtered to include only ASF‐affected countries, the distribution changed to tourism 34% (191,495), maritime crew 26.3% (148,104), residents 8.66% (48,767), maritime crew audit and consulting 6.16% (34,705), and business visits 5.89% (33,157).

Despite the low sample frequency of maritime crew categories, given the sanitary risk associated with these vessels and ASF outbreaks reported in the literature attributed to this form of AOP movement [6, 9, 10, 12], special attention was given to this category. In 2022, 182,809 people entered Brazil from ASF‐affected countries under the classifications “Maritime Crew—Long Course and Cruises” and “Maritime Crew—Audit and Consulting,” corresponding to the visa type “Transit.” Of these, 83.12% came from Asia (151,943), 16.25% from Europe (29,711), 0.59% from Africa (1087), and 68 from the Caribbean. The states that received the largest number of these crew members were SP 34.92% (63,840), RJ 15.58% (28,485), Bahia (BA) 8.49% (15,513), Paraná (PR) 7.03% (12,844), and Santa Catarina (SC) 5.69% (10,407).

**Figure 2:**
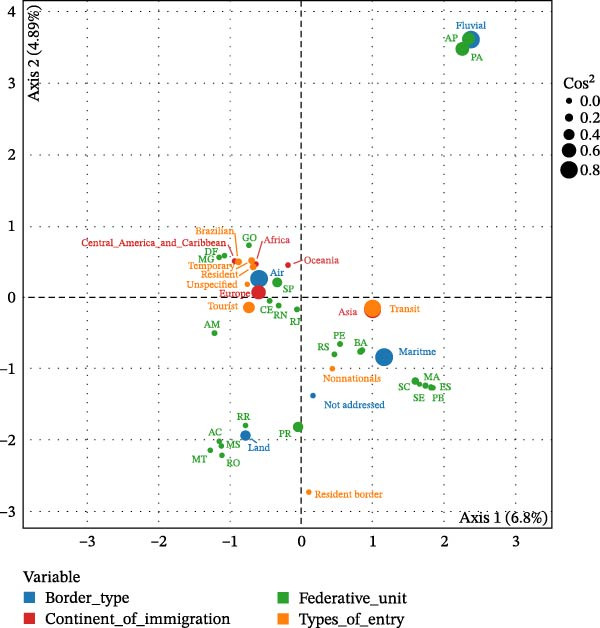
Multiple correspondence analysis of people entering Brazil in 2022 from ASF‐affected countries.

To further explore the associations between the categories of variables analyzed, such as visa type, means of entry, countries of origin, and destination states, an MCA was performed. The graphical representation of the MCA can be observed in Figure 2, where Dimension 1 is represented on the *x*‐axis and Dimension 2 on the *y*‐axis. The variables that were best represented by Dimension 1 were transit (cos^2^ = 0.72), air (cos^2^ = 0.62), Asia (cos^2^ = 0.61), maritime (cos^2^ = 0.51), and Europe (cos^2^ = 0.47). For Dimension 2, fluvial (cos^2^ = 0.54), State of Pará (PA; cos^2^ = 0.29), and Amapá (AP; cos^2^ = 0.24).

Examining the distribution of categories across Dimensions 1 and 2 revealed distinct and well‐represented profiles. States of PA (cos^2^ = 0.41) and AP (cos^2^ = 0.35), together with the representation of fluvial (cos^2^ = 0.78) entry mode, form a well‐represented group along the axes. Another well‐represented profile consists of Asian immigrants (cos^2^ = 0.63) entering via maritime (cos^2^ = 0.77) border with a “transit” visa (cos^2^ = 0.75). An additional profile of interest, with well‐represented variables, includes tourists (cos^2^ = 0.28) coming from Europe (cos^2^ = 0.47) who enter by air (cos^2^ = 0.75).

Regarding flights, according to data from the Brazilian National Civil Aviation Agency (ANAC), between 2000 and 2022, Brazil received 162,991,992 international flight passengers, averaging 7.08 million per year (± 3.02 million). During the same period, 13,691,254 passengers arrived from ASF‐affected countries.

**Figure 3:**
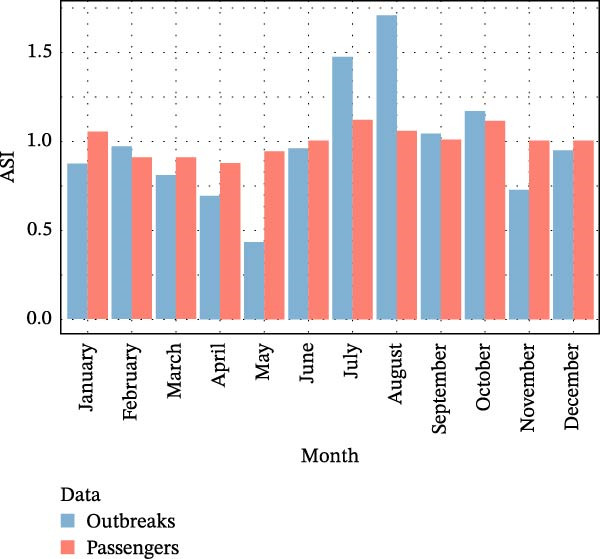
Average seasonal index of ASF outbreaks in Europe from 2010 to 2022 (including outbreaks reported in domestic pigs and wild boar) and of air passengers traveling from European ASF‐affected countries to Brazil from 2010 to 2019.

**Figure 4:**
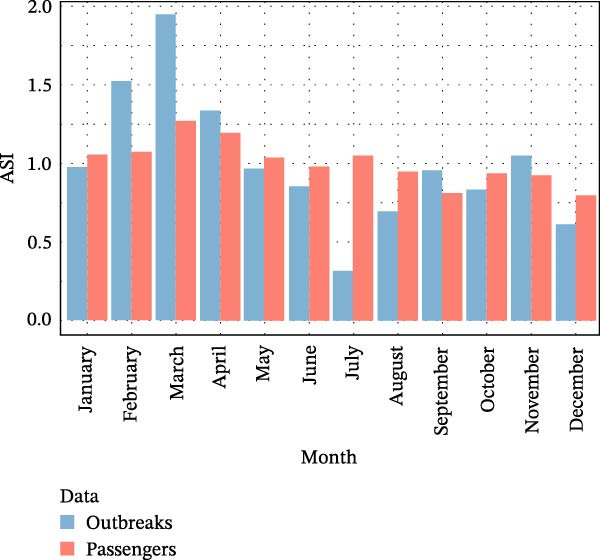
Average seasonal index of ASF outbreaks in Asia from 2018 to 2022 and of air passengers traveling from Asian ASF‐affected countries to Brazil from 2010 to 2019.

The seasonal patterns of ASF outbreaks in Europe and Asia were compared with the seasonal patterns of international passenger arrivals into Brazil from these continents. The ASI of the two series is shown in Figures 3 and 4.

In the seasonal analysis of flights originating from European ASF‐affected countries, particular attention is drawn to the months of January, July, August, and October. It is important to highlight that this seasonality coincides with the months of highest ASF outbreak incidence in Europe. The Spearman correlation between the two ASIs was 0.69, with *p* < 0.05.

The seasonality of international flights from countries with ASF‐affected countries in Asia to Brazil shows some differences compared to the European pattern, with March and April assuming greater importance. This seasonality, similar to that in Europe, mirrors the increase in ASF outbreaks in Asia, which primarily occurs in February, March, and April. The correlation between the two ASIs was 0.62, with *p* < 0.05.

#### 3.1.3. Immigrant Workers From ASF‐Affected Countries

Between 2021 and 2023, 95,245 immigrants from ASF‐affected countries were admitted to the Brazilian labor market. Of the total admissions, 88.81% (84,588) were from Haiti, 3.88% (3693) from China, 0.87% (833) from the Dominican Republic, and 0.82% (783) from Germany.

Regarding work categories, the occupation subclass “Other” accounted for the highest number of admissions, 77.22% (73,545), followed by “Poultry Slaughter” with 9.39% (8942) and “Swine Slaughter” with 5.73% (5458). The percentage distribution of each subclass for immigrants from ASF‐affected countries is presented in Table S8.

**Figure 5:**
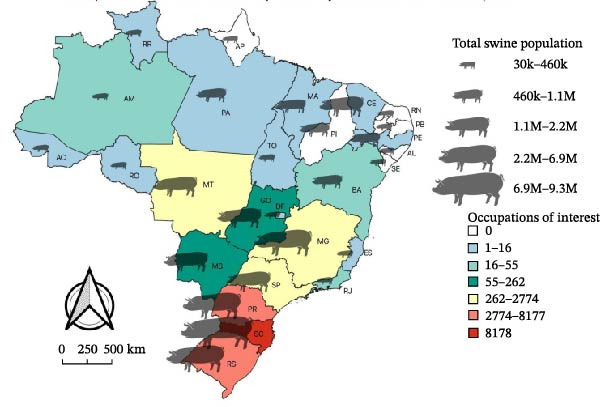
Admissions of immigrants from ASF‐affected countries into sanitary‐related occupations in the formal labor market from 2021 to 2023, and swine population by state in Brazil. *Source:* Prepared by Ailton Costa and Isadora Coelho (2025), based on data from Labor Immigration Portal (admissions from 2021 to 2023), Food and Agriculture Organization (FAO) (ASF outbreaks from 2018 to 2022), IBGE Shapefile 2024, and Brazil’s official geodetic datum (SIRGAS 2000).

Figure 5 illustrates the distribution of admissions when filtering only the categories of sanitary interest by destination. It is observed that the Southern region and the State of SP received the highest number of immigrants from ASF‐affected countries during this period. In the Southern region, the State of SC was the most important with 8178 admissions. The same figure includes the total swine population per state, aiming to show that the states receiving more immigrants from ASF‐affected countries are also the states with the highest susceptible swine population in Brazil.

In Table S9, the distribution of occupation classifications according to country of origin is shown. This subanalysis highlights the importance of Haiti, which represents 97.48% (21,154) of admissions in occupations of sanitary interest, an even higher percentage than in the general distribution, followed by the Dominican Republic with 0.6% (130), China 0.41% (88), Nigeria 0.22% (48), Bhutan 0.15% (32), South Africa 0.13% (29), and Sierra Leone 0.13% (29).

From the reclassification of the 55 subclasses of interest, some important results were obtained. For example, 452 individuals from ASF‐affected countries were admitted to work in direct contact with animals, as shown in Table 5.

**Table 5:** Admissions of immigrants from ASF‐affected countries, between 2021 and 2023, by occupation of sanitary interest.

Occupation	Count	Percentage (%)
Trade in animal products and by‐products	46	0.21
Direct contact with animals	452	2.08
Meat processing plant	16,024	73.84
Transportation and loading	1402	6.46
Other	3776	17.40
Total	21,700	100

**Figure 6:**
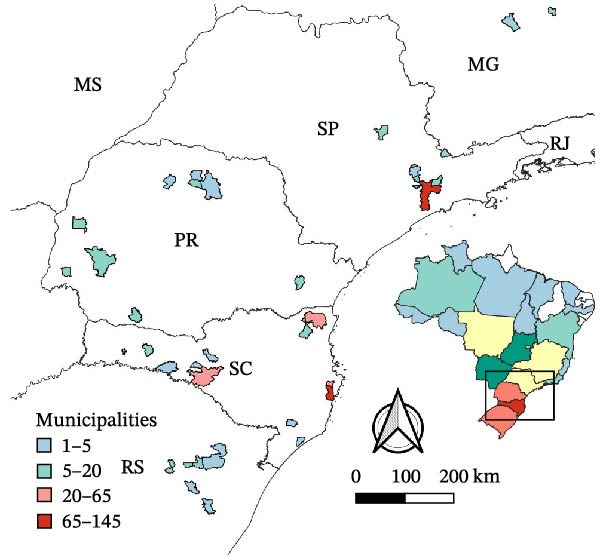
Mapping of immigrants from ASF‐affected countries to work in direct contact with animals in the formal labor market from 2021 to 2023. *Source:* Prepared by Ailton Costa and Isadora Coelho (2025), based on data from Labor Immigration Portal (admissions from 2021 to 2023), Food and Agriculture Organization (FAO) (ASF outbreaks from 2018 to 2022), IBGE Shapefile 2024, and Brazil’s official geodetic datum (SIRGAS 2000).

These workers are primarily located in the States of SP, SC, and Rio Grande do Sul (RS), corresponding to 30.31% (137), 16.37% (74), and 14.38% (65) of the category, respectively. Figure 6 presents a map showing the distribution of admissions in which the occupation allowed the worker direct contact with animals by the most frequent municipalities.

## 4. Discussion

To the best of the author’s knowledge, this is the first study to publicly provide data on the profile of key risk pathways for the introduction of ASF into Brazil, a country of major importance to the global swine industry. This study demonstrated the frequency and quantity of informally imported AOP seized from ASF‐affected countries at Brazilian borders. Additionally, it highlighted a correlation between the entry of passengers from flights originating in European and Asian ASF‐affected countries and the disease outbreaks in these countries. These data reveal critical points regarding the maintenance of Brazil’s ASF‐free status. Alongside these findings, traveler profiles, municipalities, and border entry types that may represent potential pathways for the introduction of ASF through human‐mediated movement of AOP were identified, as well as the distribution of immigrant workers from ASF‐affected countries. It is important to note that the present study does not measure viral presence in seized products nor assess the direct exposure of susceptible animals. Therefore, the risks discussed here should be interpreted as potential or conditional risks associated with pathways for virus introduction, which would only materialize if the epidemiological transmission chain was completed. Nevertheless, these findings provide valuable information to support risk‐based surveillance strategies.

More specifically, based on the FAO risk assessment, formal importation of AOP was considered to be negligible to low risk [28]. This conclusion reflects the strength of Brazilian sanitary legislation, which has prohibited the entry of AOP without an official health certificate issued by the country of origin since 1934 [40]. In addition, travelers are required to declare any AOP they are carrying and present it to customs inspection at the time of border entry. Only pork and pork products sterilized by heat treatment are permitted for import, regardless of origin [41].

In contrast, the informal importation of AOP represents a more uncertain and potentially higher‐risk pathway. Our results indicate that pork and other swine products have been seized from various countries affected by ASF in recent years. However, the profile differs according to the country and region involved in the seizure. Individuals may transport products either for trade or personal consumption. Importation for trade typically occurs in larger quantities and may involve mislabeling to circumvent bans imposed by countries with outbreaks [42]. Informal importation for personal consumption is more often associated with tourists or immigrants. Seizures from European countries such as Germany and Italy are likely more related to tourists, including Brazilians, returning with AOP as souvenirs or gifts. Conversely, countries facing severe social and food insecurity, and with swine production systems of low biosecurity, such as Haiti, raise greater sanitary concerns due to both the state of swine health and the higher likelihood that individuals are transporting AOP for personal consumption [28].

Despite the description of AOP quantities involved in seizures, Brazil does not test these products for the ASFV. This leads to uncertainty regarding whether contaminated AOPs are crossing borders [43]. Sending samples to laboratories for viral detection would be important to demonstrate the existence of the hazard and to conduct risk analyses on the introduction of the virus via AOP. Viral gene detection in seized products has been reported in several countries, including South Korea [44], Japan [45], Taiwan [46], Thailand [47], Australia [48], the Philippines [49], and Northern Ireland [50]. Beyond seizure data [26], other important factors in the epidemiological transmission chain should also be considered when evaluating the risk of ASF introduction via AOP, such as virus viability, potential contact with susceptible animals, and sufficient viral load to cause infection.

Regarding the surveillance units, results show that Tom Jobim International Airport and Guarulhos International Airport are the locations where most seizures occur. This reflects the passenger traffic volume at these airports and does not necessarily indicate that these municipalities are the final destination of the seized products. Previous studies reinforce the relevance of this work. Costard et al. [26] demonstrated that illegal imports for personal or commercial use, as well as the origin of travelers, are relevant factors in evaluating the risk of ASF introduction. No European country was considered a negligible risk; France, Germany, Italy, and the United Kingdom were high‐risk, and Spain was moderate‐risk, underscoring the importance of identifying critical routes to ASF‐free areas.

In the Brazilian context, de Melo et al. [1] described the profile of international passengers intercepted with illegal AOP at Galeão and Guarulhos airports. The most frequent nationalities were Brazil, China, Portugal, Italy, and Argentina, which corroborates our findings as Chinese, Brazilians, and Italians were also among the main nationalities involved in AOP seizures in the present study. While de Melo et al. [1] highlighted Romania and Turkey as primary risk origins, followed by Asia and Latin Europe, our results add further detail by showing that the nationality of the traveler does not always match the declared origin of the seized product. For example, whereas almost all AOP originating in China were indeed carried by Chinese travelers, approximately half of the products declared to be from Italy or Germany were seized from Brazilian passengers.

However, seizures often occur opportunistically as these products are not always the primary target of inspection. This is observed in several countries, including Brazil, where African‐origin meats, for example, are incidentally found during inspections [51]. In Brazil, the prevention of illegal entry of AOP is conducted through the International Agricultural Surveillance System (VIGIAGRO), coordinated by the MAPA. This system is responsible for inspecting animals, plants, and their products at ports, airports, and land borders to prevent the introduction of pathogens into the country [52]. Inspections include baggage screening using X‐ray equipment and physical inspections. In addition to surveillance actions, Brazilian legislation requires travelers to declare AOP and allows authorities to seize and destroy items that do not comply with sanitary requirements, and administrative penalties such as warnings or fines may be applied in cases of irregular entry [41, 52].

Given the difficulty of identifying all illegally entering AOP [53], even with inspections focused on animal health issues, it is essential to map flights and origin territories with a higher risk to guide surveillance actions. Strengthening targeted inspections based on the risk profiles identified in this study, especially those involving countries with strong ties to Brazil through tourism or labor and with circulation of high‐consequence diseases such as ASF, could improve detection. Additionally, sanitary education actions at airports and border posts are essential to inform travelers about the legal restrictions, as many are unaware of these rules [54] or do not understand their importance [26], often motivated by the desire to bring souvenirs, products from their home country, or gifts.

The seasonal alignment observed between ASF outbreaks and international passenger arrivals may reflect broader patterns of people’s movement. In Europe, both ASF outbreaks and passenger arrivals show increases during the summer months, which coincide with major holiday periods and intensified international travel. Increased human movement during these periods may facilitate the transport of AOP and other potential fomites across borders. Previous studies have suggested that the overall seasonality of ASF in Europe, particularly during winter and summer months, may be partly associated with increased human movement and activities during these periods, with the summer peak observed in both wild boar and domestic pig populations [31]. Other mechanisms have also been discussed in the literature to explain ASF seasonality. For example, the possible involvement of arthropod vectors during summer months has been hypothesized [55, 56], although current evidence remains insufficient to confirm their epidemiological role [57]. In wild boars, seasonal patterns may also be influenced by hunting activity and surveillance intensity during winter months, which can increase detection and reporting during these periods [57–59].

In Asia, the seasonal increase in outbreaks observed between February and April coincides with periods of intense human mobility associated with major cultural events such as the Chinese New Year, when travel, pork consumption, and the movement of animals and animal products increase substantially [60]. Together, these findings suggest that ASF seasonality likely reflects a combination of ecological, epidemiological, and human behavioral drivers, with increased human mobility representing one potential factor contributing to the observed temporal alignment between outbreak occurrence and international passenger flows.

Another important point is that the entry of immigrants from ASF‐affected countries in recent years shows differences in locations, border entry types, and visa types compared to the general profile. Understanding these specifics is crucial for official animal health services to more accurately target inspections of AOP entering from these countries. Since the risk is associated with the movement of people who may carry contaminated AOP, all entry routes, air, land, river, and sea, must be analyzed [43].

MCA analysis showed that border entry is an important variable in explaining the relationships among the other variables, as indicated by its cos^2^ value. Although the states are not well represented by the first two dimensions due to the heterogeneity of entry type, continent, and visa type, the clustering pattern along the dimensions resembles the geographical distribution of Brazil’s borders, linking the border entry mode with the states. For example, the category of land from the variable border type is shown to be related to northern states, such as the states of Acre (AC), Roraima (RR), and Rondonia (RO), and some central western states, such as Mato Grosso do Sul (MS) and MT. A relationship is also observed between fluvial and the states of AP and PA. Those categories are also located further from the origin, indicating a greater contribution to the deviation from independence in Dimension 1.

The descriptive results, especially for the general analysis, reflect the importance of connection centers, or hubs. In Brazil, the main hubs for air travel are Tom Jobim International Airport, in RJ, and Guarulhos International Airport in SP, while Santos Port, in SP, is the primary hub for maritime travel. Despite the large number of immigrants entering through these locations, as with AOP seizures, these points often do not represent the final destination of these individuals. However, analyses focusing on ASF‐affected countries provide important insights. Notably, there is a large volume of entries from the Northeast region of Brazil, which differs from the general analysis.

An additional important finding is that although some continents show a predominant border entry pattern, country‐specific analyses reveal important exceptions. Ukrainian immigrants, for example, enter primarily by sea. This contrasts with the general European profile. Similarly, Thailand, Malaysia, Nepal, Mongolia, Bhutan, and Cambodia predominantly enter by air, differing from the Asian pattern. Regarding land routes, the State of PR accounts for most entries via this route.

Results also highlighted the importance of transit classification profiles for individuals from ASF‐affected countries. In this context, there is a large contingent of entries via Santos and Salvador due to the significant ports in these cities. Maritime entry, especially for Asia, with many visa type granted as maritime crew, raises an important discussion point. The entry of veterinary pathogens via ship waste from foreign ports can pose a risk for disease spread in Brazil, causing economic and social losses [61]. Maritime transport historically involves precarious working conditions, including food insecurity [62], informal labor, and inadequate nutrition [63]. The poor conditions in these environments are relevant for assessing sanitary risks associated with AOP entry, as workers may bring food from home to sustain themselves during work.

Given Brazil’s importance as an exporter of AOP, maintaining herd health is crucial. While the present study characterized the frequency and profiles of AOP seizures at Brazilian borders, previous research by Michalski [61] highlights additional sanitary concerns at the Paranaguá port. In her inspection of ship cold storage, 4913.38 kg of AOP were identified, including 22.8% of meat products, 7.4% of dairy, 41.4% of fish, and 40.6% of eggs lacking origin identification. Furthermore, 18.1% of meat products were identified as containing unidentified species, and none of the inspected vessels had sanitary certification. Alarmingly, nine whole pigs and 21 half‐carcasses were found (counted by unit only), and 15% of the products were inadequately stored, with broken packaging or mixed/exposed products, including fish, meat, and vegetables. This combination of poor traceability and inadequate storage represents an even greater sanitary risk, especially considering that many of the countries of origin of these products report the presence of high‐consequence animal diseases, including FMD, classical swine fever,ASF, American foulbrood, and highly pathogenic avian influenza.

The findings align with the approach of previous risk assessment studies, such as Jurado et al. [43], which highlighted the importance of considering countries of origin, flight routes, and months when the introduction of sanitary‐important diseases is most likely. While those studies assessed the risk of introduction through these pathways, our results complement this perspective by providing detailed profiles of travelers and routes connected to Brazil. This country‐specific information can support the design of targeted preventive and control surveillance activities. Moreover, because risk is dynamic, risk‐based surveillance actions must also be flexible.

The other focus of this study, the distribution of immigrant workers from ASF‐affected countries, reveals a distinct profile from tourists. While the tourist profile captures small‐scale AOP importation risk, understanding residents’ profiles, especially from those countries, is crucial, as these data may indicate potential demand for products from these regions [26]. In this context, occupational exposure becomes an additional factor to consider. The workplace of these individuals may also represent a risk factor. If a worker carries an AOP or by‐product contaminated with the ASFV upon returning from their home country, the risk becomes even greater if their daily routine involves contact with susceptible animals [64]. Similarly, Sugiura and Haga [65] identified foreign workers as potential entry routes for the disease in Japan, particularly after visiting countries affected by the disease. In the same vein, Costard et al. [26] included the number of agricultural workers from ASF‐affected areas as a factor in their European risk analysis, highlighting the importance of understanding migratory flows in prevention strategies.

Our results allow visualization of the distribution of immigrant workers from ASF‐affected countries over the past 5 years in the context of sanitary risk associated with the ASFV introduction. Notably, a considerable number of these workers are in direct contact with livestock and swine production. Results also show that the states receiving the most of these workers are SC, PR, and RS. It is noteworthy that the Southern region is Brazil’s primary hub for swine production. According to the 2017 IBGE census, 53.63% of Brazil’s swine population is in the South. In 2022, these three states accounted for over 70% of animals slaughtered nationwide [66]. The Southern States also have the highest proportion of highly productive breeding sows, with two‐thirds of Brazil’s industrial swine establishments [67, 68].

The relevance of Haitian immigrant workers in this profile was also demonstrated, including their presence in occupations involving direct contact with production animals. As previously discussed, Haiti’s swine sector is predominantly composed of small farms with low biosecurity [69]. These findings underscore the importance of educational actions and strengthened farm biosecurity, highlighting key states and municipalities. Socio‐educational measures regarding diseases relevant to swine production are crucial for immigrant workers in contact with animals, emphasizing the potential risks associated with informal AOP importation. In Brazil, official veterinary services provide educational materials to support biosecurity in swine farms, addressing not only concerns related to ASF but also other endemic and production‐related diseases [70]. To the best of the authors’ knowledge, there is no specific nationwide continuous education program focused on ASF; instead, existing initiatives are predominantly passive, relying on the dissemination of guidelines and informational materials. These actions are complemented by partnerships with the private sector, such as the National Service for Rural Learning, which offers online courses and digital educational resources for producers and farm workers, covering topics such as management, biosafety, and animal health [71].

It is important to note that the occupational categories considered in this analysis encompass activities beyond swine production. Although ASF is restricted to suids, livestock production systems and associated service sectors often share infrastructure, transportation networks, equipment, and labor across multiple animal species. Workers, vehicles, and service providers may operate across different animal production chains, potentially acting as fomites or indirect interfaces for pathogen introduction. For this reason, a broader set of animal‐related occupational activities was included to capture potential indirect pathways of exposure. While this approach may overestimate the specific potential risk, it was adopted as a conservative strategy to avoid overlooking relevant interfaces within livestock production and animal‐product supply chains.

An important limitation of this study is the potential underestimation of risk in the international flight analysis. Although origin and layover countries were considered in the dataset used, independent connections made by passengers may not be captured. In addition, the nationality variable used to filter the ASF‐specific analysis in the STI database may introduce bias, as an immigrant’s nationality may differ from their country of origin, while the objective of this study was to relate entries from ASF‐affected countries into Brazil rather than the individual’s nationality. The analysis of AOP seizures was also limited to the most recent year with consolidated nationwide data. As Brazil continues to develop an integrated system for recording these events, future studies will be able to explore multiyear trends and temporal variability in greater detail. Finally, this analysis was based exclusively on seizure data from traveler baggage at border entry points (road and air transport) and therefore does not capture all informal importation pathways of AOP, including the potential contribution of international postal shipments to ASF introduction.

## 5. Conclusions

This study provided a comprehensive characterization of potential sanitary risk pathways related to the entry of travelers, AOP, and immigrant workers from ASF‐affected countries into Brazil. By integrating data on passenger flows, visa types, and informal AOP seizures, we identified marked heterogeneity in traveler profiles, with certain nationalities, such as Chinese, Brazilians, and Italians, representing major contributors to seized informally imported products. Products from China were almost exclusively carried by Chinese travelers, whereas a considerable proportion of products from European countries, such as Italy and Germany, were transported by Brazilian passengers. These findings underscore the importance of risk‐based sampling strategies that account for the relationship between the country of origin and the traveler’s nationality, as this varies across different nationalities. In this context, although laboratory testing of seized AOP does not directly reduce exposure, it represents a first step to determine whether contaminated products are entering the country. Although seizures are documented, the sanitary status of these products remains unknown, and generating this information is essential to support the risk assessment and guide surveillance strategies. We also observed temporal and geographic patterns in flight arrivals from ASF‐affected countries, as well as a concentration of immigrants from these countries working in sanitary‐related occupations, particularly in states with high swine densities, such as SC, RS, and SP.

These findings advance the field by providing country‐specific evidence to support risk‐based surveillance at Brazilian borders, complementing previous international risk assessments with contextual details on traveler and product profiles. They also emphasize the importance of integrated surveillance strategies that combine passenger monitoring, targeted inspections, and sanitary education efforts at airports and border posts, as well as targeted training and awareness initiatives for immigrant workers who have direct contact with animals. Future research should address current gaps, such as the absence of systematic laboratory diagnostics to verify the sanitary status of seized products, which currently limits risk assessment efforts at the federal level, and evaluate the effectiveness of existing inspection and communication strategies.

## Funding

No funding was received for this manuscript.

## Ethics Statement

This study was conducted using secondary data obtained from official databases and institutional sources. No direct animal experimentation or human subject participation was involved. In accordance with institutional policies and national regulations, formal ethical approval was not required for this type of study.

## Conflicts of Interest

The authors declare no conflicts of interest.

## Supporting Information

Additional supporting information can be found online in the Supporting Information section.

## Supporting information


**Supporting Information** Table S1: Countries reporting ASF outbreaks from 2018 to 2022, based on FAO data. Table S2: Occupation subclasses and CNAE codes considered to pose potential sanitary risk. Table S3: Animal‐origin products (kg) seized at Brazilian borders in 2023, by product type and by the 20 countries with the highest seizure numbers. Table S4: Meat and processed animal products seized (kg) at Brazilian borders from ASF‐affected countries in 2023, by Animal and Plant Health Inspection Units (MAPA). Table S5: Border entry routes of immigrants into Brazil from ASF‐affected countries, by continent of origin. Table S6: Border entry routes in Brazil in 2022, by continent and nationality of origin (ASF‐affected countries only). Table S7: Visa types of people entering Brazil in 2022, by nationality of origin (ASF‐affected countries only). Table S8: Admissions to the formal labor market in Brazil from 2021 to 2023, by occupation subclasses, for immigrants from ASF‐affected countries. Table S9: Admissions to the formal labor market in Brazil from 2021 to 2023, by country of origin and occupations of sanitary interest, for immigrants from ASF‐affected countries.

## Data Availability

The data that support the findings of this study are available from the corresponding author upon reasonable request.
