# Supplementary material for: Characterization of Potential Risk Pathways for African Swine Fever Introduction Into Brazil
Source: Transbound Emerg Dis. 2026 May 21;2026:4187659. doi: 10.1155/tbed/4187659 (PMC13191771; doi:10.1155/tbed/4187659)
Supplement: Supplementary file 1 — Supporting Information Table S1: Countries reporting ASF outbreaks from 2018 to 2022, based on FAO data. Table S2: Occupation subclasses and CNAE codes considered to pose potential sanitary risk. Table S3: Animal‐origin products (kg) seized at Brazilian borders in 2023, by product type and by the 20 countries with the highest seizure numbers. Table S4: Meat and processed animal products seized (kg) at Brazilian borders from ASF‐affected countries in 2023, by Animal and Plant Health Inspection Units (MAPA). Table S5: Border entry routes of immigrants into Brazil from ASF‐affected countries, by continent of origin. Table S6: Border entry routes in Brazil in 2022, by continent and nationality of origin (ASF‐affected countries only). Table S7: Visa types of people entering Brazil in 2022, by nationality of origin (ASF‐affected countries only). Table S8: Admissions to the formal labor market in Brazil from 2021 to 2023, by occupation subclasses, for immigrants from ASF‐affected countries. Table S9: Admissions to the formal labor market in Brazil from 2021 to 2023, by country of origin and occupations of sanitary interest, for immigrants from ASF‐affected countries. [file TBED-2026-4187659-s001.docx]

**Supplementary Material**

**S1. Countries reporting ASF outbreaks from 2018 to 2022, based on FAO data.**

| 1. Germany | 19. Greece | 37. Republic of Korea |
| --- | --- | --- |
| 2. Arunachal Pradesh | 20. Haiti | 38. Republic of Moldova |
| 3. Belgium | 21. Hong Kong | 39. Lao People’s Democratic Republic |
| 4. Belarus | 22. Hungary | 40. Dominican Republic |
| 5.Bosnia and Herzegovina | 23. India | 41. Czech Republic |
| 6. Bulgaria | 24. Indonesia | 42. Romania |
| 7. Bhutan | 25. Italy | 43. Serbia |
| 8. Cambodia | 26. Kenya | 44. Sierra Leone |
| 9. Chad | 27. Latvia | 45. Thailand |
| 10. China | 28. Lithuania | 46. Timor-Leste |
| 11. Cingapura | 29. North Macedonia | 47. Tunisia |
| 12. South Korea | 30. Malasya | 48. Ukraine |
| 13. Côte d'Ivoire | 31. Namibia | 49. United Republic of Tanzania |
| 14. Croatia | 32. Nepal | 50. Vietnam |
| 15. Slovakia | 33. Nigeria | 51. Zambia |
| 16. Estonia | 34. Papua New Guinea | 52. Zimbabwe |
| 17. Russian Federation | 35. Poland |  |
| 18. Philippines | 36. Central African Republic |  |

**S2. Occupation subclasses and CNAE codes considered to pose potential sanitary risk.**

| **Code** | **Subclass** | **Code** | **Subclass** |
| --- | --- | --- | --- |
| 151201 | Cattle raising for meat | 1013902 | Preparation of byproducts from slaughter |
| 151202 | Dairy cattle raising | 3314711 | Agricultural machinery maintenance |
| 151203 | Other cattle raising | 3314712 | Tractor maintenance |
| 152101 | Buffalo raising | 4623101 | Wholesale trade of live animals |
| 152102 | Horse raising | 4623102 | Wholesale trade of non-edible POAs |
| 152103 | Donkeys and mules raising | 4634601 | Trade in beef and pork |
| 153901 | Goat raising | 4634602 | Trade in poultry meat |
| 153902 | Sheep raising | 4634699 | Trade in other meat |
| 154700 | Swine raising | 4722901 | Butcher shops |
| 155501 | Broiler chicken raising | 4771704 | Trade in veterinary medicines |
| 155502 | Chick production | 4789004 | Trade in live animals and pet food |
| 155503 | Raising other Poultry | 4911600 | Rail freight transport |
| 155504 | Poultry raising | 4930201 | Road freight transport |
| 155505 | Egg production | 5011401 | Coastal shipping |
| 162801 | Artificial insemination | 5012201 | Long-distance maritime transport |
| 162802 | Shearing of sheep | 5021101 | Municipal navigation transport |
| 162803 | Animal management | 5021102 | Intermunicipal, interstate, and international navigation transport |
| 162899 | Livestock support | 5120000 | Air cargo transport |
| 170900 | Hunting | 5212500 | Loading and unloading |
| 1011201 | Cattle slaughter | 5222200 | Road and rail terminals |
| 1011202 | Horse slaughter | 5250801 | Dispatch agency |
| 1011203 | Sheep and goat slaughter | 5250802 | Customs brokers |
| 1011204 | Buffalo slaughter | 5250803 | Freight forwarding |
| 1011205 | Slaughterhouse | 5250804 | Cargo transport logistics |
| 1012101 | Poultry slaughter | 7490103 | Agricultural consultancy |
| 1012103 | Swine slaughter | 7500100 | Veterinary activities |
| 1012104 | Swine slaughter | 7820500 | Temporary labor leasing |
| 1013901 | Meat product manufacturing |  | |

**S3. Animal-origin products (kg) seized at Brazilian borders in 2023, by product type and by the 20 countries with the highest seizure numbers.**

| **Country of origin** | **Meat and sausages** | **Dairy products** | **Eggs** | **Fish** | **Beekeeping** | **Total** | **Total (%)** |
| --- | --- | --- | --- | --- | --- | --- | --- |
| Argentina | 10,567.07 | 1,205.71 | 1,485.48 | 784,69 | 16,91 | 14,059.86 | 43,27 |
| Bolivia | 1,870.57 | 2,404.99 | 512,6 | 106,9 | 89,02 | 4,984.08 | 15,34 |
| Portugal | 2,155.96 | 126,46 | 1,7 | 809,23 | 93,23 | 3,186.58 | 9,81 |
| Paraguay | 1,278.98 | 290,79 | 498,03 | 138,71 | 222,26 | 2,428.77 | 7,48 |
| China | 485,11 | 0,51 | 43,34 | 1,387.49 | 9,45 | 1,925.9 | 5,93 |
| Nigeria | 33,42 | 0 | 0 | 945,61 | 23,32 | 1,002.35 | 3,09 |
| Venezuela | 101,74 | 249,76 | 3,14 | 252,91 | 29,6 | 637,15 | 1,96 |
| Spain | 579,14 | 11,3 | 0 | 16,9 | 6,11 | 613,45 | 1,89 |
| Italy | 410,79 | 133 | 0,18 | 14,7 | 8,23 | 566,89 | 1,74 |
| Angola | 55,4 | 0,65 | 0 | 271,01 | 1,88 | 328,94 | 1,01 |
| Haiti | 10,8 | 0 | 0 | 223,88 | 8,47 | 243,15 | 0,75 |
| Peru | 71,57 | 105,9 | 0,83 | 19,55 | 19,89 | 217,74 | 0,67 |
| USA | 136,79 | 12,21 | 1,1 | 38,66 | 20,13 | 208,89 | 0,64 |
| France | 95,04 | 30,36 | 0 | 1,17 | 6,75 | 133,32 | 0,41 |
| Colombia | 92,71 | 14,64 | 0,8 | 3,25 | 1,14 | 112,54 | 0,35 |
| South Korea | 25,12 | 0,54 | 0,14 | 81,29 | 0 | 107,09 | 0,33 |
| Senegal | 19,64 | 0 | 0 | 81,88 | 2,3 | 103,82 | 0,32 |
| Guiana | 13,84 | 1,5 | 0 | 83,58 | 0 | 98,92 | 0,31 |
| **Total** | **18,658.58** | **4,742.54** | **2,570.74** | **5,834.82** | **683,94** | **32,490.62** | **100** |

**S4. Meat and processed animal products seized (kg) at Brazilian borders from ASF-affected countries in 2023, by Animal and Plant Health Inspection Units (MAPA).**

| **Inspection Unit** | **Total meat and processed products (kg)** | **%** | **Inspection Unit** | **Total meat and processed products (kg)** | **%** |
| --- | --- | --- | --- | --- | --- |
| AM/VIGI-MAO | 17,95 | 1,50 | RJ/VIGI-GIG | 312,54 | 26,12 |
| BA/VIGI-SSA | 40,88 | 3,42 | RN/VIGI-NAT | 54,24 | 4,53 |
| CE/VIGI-FOR | 59,15 | 4,94 | RS/VIGI-POA | 2,13 | 0,18 |
| DF/VIGI-BSB | 47,36 | 3,96 | SP/VIGI-GRU | 613,32 | 51,26 |
| MG/VIGI-CNF | 33,29 | 2,78 | SP/VIGI-VCP | 14,65 | 1,22 |
| PA/VIGI-BEL | 0,9 | 0,08 | **Total** | **1,196.41** | **100** |

**S5. Border entry routes of immigrants into Brazil from ASF-affected countries, by continent of origin.**

| **Continent** | **Air** | **%** | **River** | **%** | **Sea** | **%** | **Land** | **%** | **Total** |
| --- | --- | --- | --- | --- | --- | --- | --- | --- | --- |
| Africa | 15,452 | 91,05 | 89 | 0,52 | 1,140 | 6,72 | 289 | 1,70 | 16,970 |
| Central America and Caribbean | 12,071 | 93,00 | 85 | 0,65 | 56 | 0,43 | 767 | 5,91 | 12,979 |
| Asia | 67,097 | 31,38 | 17,934 | 8,39 | 127,196 | 59,48 | 1,616 | 0,76 | 213,843 |
| Europe | 267,319 | 83,71 | 4,422 | 1,38 | 25,555 | 8,00 | 22,052 | 6,91 | 319,350 |
| Oceania | 17 | 100 | 0 | 0 | 0 | 0 | 0 | 0 | 17 |
| **Total** | **361,956** | **64,27** | **22,530** | **4,00** | **153,947** | **27,34** | **24,724** | **4,39** | **563,159** |

## **S6. Border entry routes in Brazil in 2022, by continent and nationality of origin (ASF-affected countries only).**

| **Europe** | | | | | | | | | | |
| --- | --- | --- | --- | --- | --- | --- | --- | --- | --- | --- |
| **Nationality** | **Air** | **%** | **River** | **%** | **Sea** | **%** | **Land** | **%** | **Total** |  |
| Germany | 104,214 | 88,70 | 205 | 0,17 | 3,075 | 2,62 | 9,989 | 8,50 | 117,485 |  |
| Belgium | 15,859 | 89,79 | 46 | 0,26 | 141 | 0,80 | 1,617 | 9,15 | 17,663 |  |
| Belarus | 1,022 | 86,98 | 9 | 0,77 | 49 | 4,17 | 95 | 8,09 | 1,175 |  |
| Bulgaria | 1,864 | 55,33 | 350 | 10,39 | 717 | 21,28 | 438 | 13,00 | 3,369 |  |
| Croatia | 2,330 | 59,26 | 170 | 4,32 | 1,362 | 34,64 | 70 | 1,78 | 3,932 |  |
| Slovakia | 1542 | 74,64 | 22 | 1,06 | 56 | 2,71 | 446 | 21,59 | 2,066 |  |
| Estonia | 689 | 85,06 | 4 | 0,49 | 47 | 5,80 | 70 | 8,64 | 810 |  |
| Greece | 4,492 | 55,59 | 319 | 3,95 | 2,673 | 33,08 | 597 | 7,39 | 8,081 |  |
| Hungary | 2,608 | 83,40 | 5 | 0,16 | 87 | 2,78 | 427 | 13,66 | 3,127 |  |
| Italy | 101,327 | 93,08 | 112 | 0,10 | 2,962 | 2,72 | 4,457 | 4,09 | 108,858 |  |
| Latvia | 1,168 | 65,00 | 19 | 1,06 | 451 | 25,10 | 159 | 8,85 | 1,797 |  |
| Lithuania | 1,645 | 83,97 | 31 | 1,58 | 154 | 7,86 | 129 | 6,58 | 1,959 |  |
| Poland | 12,135 | 76,36 | 516 | 3,25 | 1,701 | 10,70 | 1,539 | 9,68 | 15,891 |  |
| Czech Republic | 3,234 | 83,42 | 30 | 0,77 | 29 | 0,75 | 584 | 15,06 | 3,877 |  |
| Romania | 5,159 | 60,31 | 284 | 3,32 | 2,105 | 24,61 | 1,006 | 11,76 | 8,554 |  |
| Serbia | 2,071 | 90,56 | 24 | 1,05 | 120 | 5,25 | 72 | 3,15 | 2,287 |  |
| Ukraine | 5,960 | 32,36 | 2,276 | 12,36 | 9,826 | 53,35 | 357 | 1,94 | 18,419 |  |
| **Total** | **267,319** | **83,71** | **4,422** | **1,38** | **25,555** | **8,00** | **22,052** | **6,91** | **319,350** |  |
| **Asia** | | | | | | | | | | |
| **Nationality** | **Air** | **%** | **River** | **%** | **Sea** | **%** | **Land** | **%** | **Total** |  |
| Philippines | 21,123 | 19,18 | 12,127 | 11,01 | 76,787 | 69,72 | 95 | 0,09 | 110,132 |  |
| India | 20,992 | 40,46 | 3,210 | 6,19 | 27,371 | 52,75 | 313 | 0,60 | 51,886 |  |
| China | 15,346 | 47,01 | 1,677 | 5,14 | 14,911 | 45,68 | 710 | 2,17 | 32,644 |  |
| Indonesia | 2,851 | 30,57 | 522 | 5,60 | 5,885 | 63,10 | 69 | 0,74 | 9,327 |  |
| Thailand | 2,585 | 78,17 | 123 | 3,72 | 480 | 14,51 | 119 | 3,60 | 3,307 |  |
| Malasya | 2,267 | 78,20 | 18 | 0,62 | 347 | 11,97 | 267 | 9,21 | 2,899 |  |
| Vietnam | 588 | 26,17 | 256 | 11,39 | 1,371 | 61,01 | 32 | 1,42 | 2,247 |  |
| Nepal | 1,125 | 96,73 | 1 | 0,09 | 35 | 3,01 | 2 | 0,17 | 1,163 |  |
| Mongólia | 140 | 90,32 |  | 0 | 6 | 3,87 | 9 | 5,81 | 155 |  |
| Bhutan | 53 | 100 |  | 0 |  | 0 |  | 0 | 53 |  |
| Cambodia | 27 | 90,00 |  | 0 | 3 | 10,00 |  | 0 | 30 |  |
| **Total** | **67,097** | **31,38** | **17,934** | **8,39** | **127,196** | **59,48** | **1,616** | **0,76** | **213,843** |  |
| **Central America** | | | | | | | | | | |
| **Nationality** | **Air** | **%** | **River** | **%** | **Sea** | **%** | **Land** | **%** | **Total** |  |
| Haiti | 6,675 | 96,52 | 35 | 0,51 | 4 | 0,06 | 202 | 2,92 | 6,916 |  |
| Dominican Republic | 5,396 | 89,00 | 50 | 0,82 | 52 | 0,86 | 565 | 9,32 | 6,063 |  |
| **Total** | **12,071** | **93,00** | **85** | **0,65** | **56** | **0,43** | **767** | **5,91** | **12,979** |  |
| **Africa** | | | | | | | | | | |
| **Nationality** | **Air** | **%** | **River** | **%** | **Sea** | **%** | **Land** | **%** | **Total** |  |
| South Africa | 6,660 | 88,19 | 69 | 0,91 | 621 | 8,22 | 202 | 2,67 | 7,552 |  |
| Nigeria | 5,041 | 98,75 | 8 | 0,16 | 32 | 0,63 | 24 | 0,47 | 5,105 |  |
| Tunisia | 1,720 | 93,22 | 4 | 0,22 | 78 | 4,23 | 43 | 2,33 | 1,845 |  |
| Kenya | 1,245 | 80,53 | 7 | 0,45 | 286 | 18,50 | 8 | 0,52 | 1,546 |  |
| Namibia | 187 | 59,55 | 1 | 0,32 | 117 | 37,26 | 9 | 2,87 | 314 |  |
| Ivory Coast | 296 | 99,66 |  | 0 | 1 | 0,34 |  | 0 | 297 |  |
| Sierra Leone | 147 | 98,66 |  | 0 | 2 | 1,34 |  | 0 | 149 |  |
| Zambia | 126 | 99,21 |  | 0 | 1 | 0,79 |  | 0 | 127 |  |
| Chad | 30 | 85,71 |  | 0 | 2 | 5,71 | 3 | 8,57 | 35 |  |
| **Total** | **15,452** | **91,05** | **89** | **0,52** | **1,140** | **6,72** | **289** | **1,70** | **16,970** |  |

Source: Prepared by the author (2024),

## **S7. Visa types of people entering Brazil in 2022, by nationality of origin (ASF-affected countries only).**

| **Nationality** | **Resident** | **%** | **Temporary** | **%** | **Transit** | **%** | **Tourist** | **%** | **Total** |
| --- | --- | --- | --- | --- | --- | --- | --- | --- | --- |
| South Africa | 707 | 9,36 | 1,306 | 17,29 | 2,004 | 26,54 | 3,505 | 46,41 | 7,552 |
| Germany | 11,028 | 9,39 | 12,479 | 10,62 | 14,769 | 12,57 | 73,126 | 62,24 | 117,485 |
| Belgium | 1,980 | 11,21 | 2,014 | 11,40 | 1,310 | 7,42 | 11,154 | 63,15 | 17,663 |
| Belarus | 46 | 3,91 | 83 | 7,06 | 300 | 25,53 | 744 | 63,32 | 1,175 |
| Bulgaria | 196 | 5,82 | 251 | 7,45 | 1,451 | 43,07 | 1,449 | 43,01 | 3,369 |
| Bhutan | 4 | 7,55 | 11 | 20,75 | 35 | 66,04 | 3 | 5,66 | 53 |
| Cambodia | 5 | 16,67 | 8 | 26,67 | 4 | 13,33 | 12 | 40,00 | 30 |
| Chad | 8 | 22,86 | 6 | 17,14 | 4 | 11,43 | 17 | 48,57 | 35 |
| China | 5,133 | 15,72 | 4,103 | 12,57 | 17,323 | 53,07 | 6,056 | 18,55 | 32,644 |
| Côte d'Ivoire | 87 | 29,29 | 69 | 23,23 | 6 | 2,02 | 134 | 45,12 | 297 |
| Croatia | 443 | 11,27 | 405 | 10,30 | 2,037 | 51,81 | 1,018 | 25,89 | 3,932 |
| Slovakia | 97 | 4,70 | 237 | 11,47 | 233 | 11,28 | 1,478 | 71,54 | 2,066 |
| Estonia | 55 | 6,79 | 88 | 10,86 | 78 | 9,63 | 574 | 70,86 | 810 |
| Philippines | 1,559 | 1,42 | 1,612 | 1,46 | 105,404 | 95,71 | 1,554 | 1,41 | 110,132 |
| Greece | 367 | 4,54 | 797 | 9,86 | 4005 | 49,56 | 2,799 | 34,64 | 8,081 |
| Haiti | 2,988 | 43,20 | 3,460 | 50,03 | 12 | 0,17 | 438 | 6,33 | 6,916 |
| Hungary | 241 | 7,71 | 291 | 9,31 | 341 | 10,91 | 2,127 | 68,02 | 3,127 |
| India | 3,170 | 6,11 | 3,439 | 6,63 | 40,708 | 78,46 | 4,558 | 8,78 | 51,886 |
| Indonesia | 278 | 2,98 | 400 | 4,29 | 7,862 | 84,29 | 782 | 8,38 | 9,327 |
| Italy | 16,130 | 14,82 | 9,977 | 9,17 | 9,156 | 8,41 | 49,854 | 45,80 | 108,858 |
| Latvia | 137 | 7,62 | 157 | 8,74 | 729 | 40,57 | 757 | 42,13 | 1,797 |
| Lithuania | 172 | 8,78 | 193 | 9,85 | 367 | 18,73 | 1,181 | 60,29 | 1,959 |
| Malasya | 268 | 9,24 | 499 | 17,21 | 1,059 | 36,53 | 1,071 | 36,94 | 2,899 |
| Mongolia | 21 | 13,55 | 15 | 9,68 | 22 | 14,19 | 97 | 62,58 | 155 |
| Namibia | 20 | 6,37 | 166 | 52,87 | 16 | 5,10 | 112 | 35,67 | 314 |
| Nepal | 884 | 76,01 | 37 | 3,18 | 82 | 7,05 | 160 | 13,76 | 1,163 |
| Nigeria | 3,498 | 68,52 | 443 | 8,68 | 98 | 1,92 | 1,053 | 20,63 | 5,105 |
| Papua New Guinea | 2 | 11,76 | 1 | 5,88 | 13 | 76,47 | 1 | 5,88 | 17 |
| Poland | 1,872 | 11,78 | 1,794 | 11,29 | 3,327 | 20,94 | 8,577 | 53,97 | 15,891 |
| Kenya | 73 | 4,72 | 341 | 22,06 | 819 | 52,98 | 313 | 20,25 | 1,546 |
| Dominican Republic | 584 | 9,63 | 826 | 13,62 | 235 | 3,88 | 4,411 | 72,75 | 6,063 |
| Czech Republic | 240 | 6,19 | 435 | 11,22 | 358 | 9,23 | 2,790 | 71,96 | 3,877 |
| Romania | 510 | 5,96 | 695 | 8,12 | 3,635 | 42,49 | 3,685 | 43,08 | 854 |
| Sierra Leone | 53 | 35,57 | 56 | 37,58 | 6 | 4,03 | 34 | 22,82 | 149 |
| Serbia | 159 | 6,95 | 197 | 8,61 | 778 | 34,02 | 1,141 | 49,89 | 2,287 |
| Thailand | 210 | 6,35 | 321 | 9,71 | 1,870 | 56,55 | 884 | 26,73 | 3,307 |
| Tunisia | 103 | 5,58 | 238 | 12,90 | 672 | 36,42 | 831 | 45,04 | 1,845 |
| Ukraine | 490 | 2,66 | 668 | 3,63 | 14,532 | 78,90 | 2,715 | 14,74 | 18,419 |
| Vietnam | 145 | 6,45 | 114 | 5,07 | 1,714 | 76,28 | 271 | 12,06 | 2,247 |
| Zambia | 37 | 29,13 | 43 | 33,86 | 18 | 14,17 | 29 | 22,83 | 127 |

## **S8. Admissions to the formal labor market in Brazil from 2021 to 2023, by occupation subclasses, for immigrants from ASF-affected countries.**

| **Ocuppation subclasses** | **Admissions** | **%** |
| --- | --- | --- |
| Other | 73,545 | 77,22 |
| Poultry slaughter | 8,942 | 9,39 |
| Swine slaughter | 5,458 | 5,73 |
| Temporary labor leasing | 3,380 | 3,55 |
| Loading and unloading | 737 | 0,77 |
| Cattle slaughter | 675 | 0,71 |
| Preparation of byproducts from slaughter | 498 | 0,52 |
| Butcher shops | 439 | 0,46 |
| Meat product manufacturing | 376 | 0,39 |
| Cargo transport logistics | 323 | 0,34 |
| Road freight transport | 263 | 0,28 |
| Egg production | 134 | 0,14 |
| Trade in live animals and pet food | 63 | 0,07 |
| Freight forwarding | 59 | 0,06 |
| Chick production | 46 | 0,05 |
| Cattle raising for meat | 43 | 0,05 |
| Poultry raising | 42 | 0,04 |
| Trade in beef and pork | 37 | 0,04 |
| Livestock support | 36 | 0,04 |
| Swine raising | 23 | 0,02 |
| Broiler chicken raising | 14 | 0,01 |
| Veterinary activities | 12 | 0,01 |
| Trade in veterinary medicines | 12 | 0,01 |
| Animal management | 12 | 0,01 |
| Dairy cattle raising | 8 | 0,01 |
| Raising other poultry | 8 | 0,01 |
| Dispatch agency | 7 | 0,01 |
| Rail freight transport | 6 | 0,01 |
| Agricultural consultancy | 5 | 0,01 |
| Slaughterhouse | 5 | 0,01 |
| Horse slaughter | 4 | 0 |
| Agricultural machinery maintenance | 4 | 0 |
| Tractor maintenance | 4 | 0 |
| Road and rail terminals | 4 | 0 |
| Sheep and goat slaughter | 3 | 0 |
| Wholesale trade in non-food POA | 3 | 0 |
| Trade in poultry meat | 3 | 0 |
| Trade in other meat | 3 | 0 |
| Horse raising | 2 | 0 |
| Sheep raising | 2 | 0 |
| Intercity, interstate, and international shipping | 2 | 0 |
| Wholesale trade in live animals | 1 | 0 |
| Buffalo raising | 1 | 0 |
| Long-distance maritime transport | 1 | 0 |
| **Total** | **95,245** | **100** |

## **S9. Admissions to the formal labor market in Brazil of immigrant workers from ASF-affected countries from 2021 and 2023, by country of origin and occupations of sanitary interest.**

| **Country of origin** | **Trade in POA** | **Direct contact with animals** | **Slaughterhouse** | **Others** | **Transport and cargo** | **Total** |
| --- | --- | --- | --- | --- | --- | --- |
| Haiti | 44 | 414 | 15,778 | 3,616 | 1,302 | 21,154 |
| Dominican Republic | | 1 | 106 | 19 | 4 | 130 |
| China |  | 4 | 7 | 25 | 52 | 88 |
| Nigeria |  |  | 22 | 24 | 2 | 48 |
| Bhutan |  | 7 | 23 | 1 | 1 | 32 |
| Sierra Leone | | 1 | 28 |  |  | 29 |
| África do Sul | | 3 | 19 | 6 | 1 | 29 |
| Germany | | 8 | 3 | 8 | 6 | 25 |
| South Korea | |  |  | 13 | 11 | 24 |
| Côte d'Ivoire | |  | 3 | 15 | 1 | 19 |
| Italy |  | 2 | 2 | 10 | 3 | 17 |
| Tunisia |  |  | 10 | 4 | 2 | 16 |
| Kenya |  | 1 | 7 | 1 | 3 | 12 |
| Belarus | | 1 | 5 | 5 |  | 11 |
| Philippines |  |  | 1 | 6 | 1 | 8 |
| Cambodia |  | 1 | 3 | 1 | 1 | 6 |
| Vietnam | 1 |  | 1 | 2 | 2 | 6 |
| Belgium | 1 | 1 |  | 2 | 1 | 5 |
| Ukraine |  |  |  | 5 |  | 5 |
| Singapore | | 3 | 1 |  |  | 4 |
| Central African Republic | | | 1 |  | 3 | 4 |
| Thailand | | 1 | 1 |  | 2 | 4 |
| Rússia |  | 2 |  | 2 |  | 4 |
| Romania |  |  | 1 | 1 | 1 | 3 |
| Tanzânia | |  |  | 2 | 1 | 3 |
| Croatia |  | 1 |  | 1 |  | 2 |
| Indonesia | | 1 |  | 1 |  | 2 |
| Poland |  |  |  | 2 |  | 2 |
| Serbia |  |  |  | 2 |  | 2 |
| Hungary |  |  | 1 |  |  | 1 |
| Laos |  |  | 1 |  |  | 1 |
| Estonia |  |  |  | 1 |  | 1 |
| Latvia |  |  |  | 1 |  | 1 |
| Mongólia | |  |  |  | 1 | 1 |
| Myamanar | |  |  |  | 1 | 1 |
| **Total** | **46** | **452** | **16,024** | **3,776** | **1,402** | **21,700** |
